# Proportion of Traditional Uvulectomy and Associated Factors Among Caregivers of Children Under Five‐Years Old Visiting Tibebe Ghion Specialized Hospital, Northwest Ethiopia: A Cross‐Sectional Study

**DOI:** 10.1002/hsr2.70675

**Published:** 2025-04-18

**Authors:** Gebiyaw Wudie Tsegaye, Martha Tuji, Shitahun Fentie

**Affiliations:** ^1^ Department of Epidemiology and Biostatistics, College of Medicine and Health Sciences Bahir Dar University Bahir Dar Ethiopia; ^2^ Department of Pediatrics and Child Health, College of Medicine and Health Sciences Bahir Dar University Bahir Dar Ethiopia

**Keywords:** caregivers, children under‐five, proportion, TGSH, traditional uvulectomy

## Abstract

**Background and Aims:**

Traditional uvulectomy is prevalent in Ethiopia, and is most commonly performed on children under 5 years old with a particular focus on infants under 6 months. The practice continues to be a significant issue in Ethiopia. This study aims to assess the proportion of traditional uvulectomy and its associated factors among caregivers of children under 5 years old visiting Tibebe Ghion Specialized Hospital (TGSH) in Northwest Ethiopia in 2023.

**Methods:**

An institutional‐based cross‐sectional study was conducted from October 1 to December 30, 2023, at TGSH. A total of 402 caregivers with children under 5 years old were selected using a systematic random sampling technique. Data were analyzed using SPSS version 23. Both Bivariable and multivariable analyses were performed to identify associated factors. Variables with a *p*‐value less than 0.25 in the bivariable Logistic regression analysis were included in the multivariable analysis. Odds ratios with 95% confidence intervals and *p*‐values < 0.05 were used to determine significant associations.

**Results:**

The proportion of traditional uvulectomy was 46%. Approximately 97.3% of the procedures were performed on children under 6 months of age. Caregivers who could not read and write were 1.85 times more likely to practice uvulectomy than those who could read and write (AOR: 1.85, 95% CI: 1.39–6.27). Caregivers from rural areas were 2.81 times more likely to practice than caregivers from urban areas (AOR: 2.81, 95% CI: 1.63–5.68), those without ANC follow‐up were 5.20 times more likely to practice uvulectomy compared to caregivers who had ANC follow up (AOR: 5.20, 95% CI: 2.06–8.63), those had no information about harmful effects of traditional uvulectomy were 2.43 times more likely to practice uvulectomy than their counterparts (AOR: 2.43, 95% CI: 1.24–4.55), and those who had witnessed good outcome of traditional uvulectomy were 6.05 times more likely to practice uvulectomy (AOR: 6.05, 95% CI: 3.64–12.11).

**Conclusion:**

The Proportion of traditional uvulectomy remains high in the study area, and it is associated with factors such as illiteracy, lack of ANC follow‐up, rural residency, lack of awareness about the harmful effects of uvulectomy, and witnessing positive outcomes. Immediate actions should include increasing awareness through ANC counseling and educating caregivers on the risk of uvulectomy. Long‐term solutions could involve policy changes and community‐based interventions aimed at reducing the practice and its associated risks.

**Abbreviations**
ANC: antenatal care
CI: confidence interval
ENT: ear, nose, and throat
E.C: Ethiopian calendar
HIV: human immune deficiency virus
HTPs: harmful traditional practices
IMNCI: integrated management for neonatal and child illness
NICU: neonatal intensive care unit
PCH: pediatrics and child health
SPSS: statistical package for social science
TGSH: Tibebe Ghion specialized hospital

## 1 Introduction

The uvula is a small, soft tissue structure that hangs from the back of the mouth above the throat, between the two tonsils. It serves several functions, including preventing aspiration, lubricating the oropharyngeal mucosa, aiding in language communication, boosting immunological function, and preventing regurgitation of breast milk [1].

Uvulectomy involves the partial or complete removal of the uvula through surgery [2]. This procedure can be part of modern medicine to address medical conditions such as snoring, or it can be part of traditional medicine to treat conditions like sore throat, which may be dangerous [3]. Traditional uvulectomy, performed by nonphysician healers, is an unsupervised practice that can be hazardous, potentially leading to the spread of infectious diseases such as HIV and hepatitis [4, 5]. In Ethiopia, traditional uvulectomy has been associated with high morbidity and occasional mortality, with complications including hemorrhage and sepsis [6].

The practice is widespread in Africa, often rooted in cultural beliefs that an elongated uvula causes throat problems. It is performed in response to various symptoms, including sore throat, chronic cough, vomiting, diarrhea, rejection of breast milk, growth retardation, and fever [7, 8]. The procedure is most common in children under 5 years old, with a peak incidence in those aged 0–12 months [9]. It is more prevalent in rural areas than in urban centers [7, 10].

To address this issue, Ethiopia has implemented a national strategy to eradicate harmful traditional practices, including traditional uvulectomy, by 2025. The Integrated Management of Neonatal and Childhood Illness (IMNCI) strategy includes preventive and curative interventions to improve practices in health facilities and at home, aiming to reduce harmful traditional practices [11, 12].

Despite these national efforts, traditional uvulectomy remains prevalent, particularly in the northern regions of Ethiopia. Cultural beliefs and limited access to education continue to perpetuate the practice. A national survey conducted in Ethiopia in 2005 indicated that approximately 84.3% of the general population practiced uvulectomy, with 48.6% aware of its harmful effects [1]. Studies have shown that parents and traditional practitioners often believe the uvula causes throat disorders, leading to its removal [13, 14]. However, this practice may not resolve the underlying health issues and can result in the persistence of symptoms despite the procedure [8].

Complications of traditional uvulectomy are severe and include HIV, hepatitis, anemia, hemorrhage, sepsis, jaundice, and other life‐threatening conditions, often due to unsanitary practice and inadequate medical supervision [7, 15, 16]. Despite these risks, traditional practitioners continue to perform uvulectomy due to cultural beliefs and inadequate formal education [17, 18]. The practice persists in developing countries, often influenced by socioeconomic factors and lack of awareness [11, 19]. Efforts to eliminate traditional uvulectomy should be intensified, and this study aims to provide data on the proportion of traditional uvulectomy and its associated factors among caregivers of children under 5 years old visiting Tibebe Ghion Specialized Hospital (TGSH) in Northwest Ethiopia. Understanding these factors is crucial for planning effective interventions and achieving the national goal of ending harmful traditional practices.

### 1.1 General Objective

To assess the proportion of traditional uvulectomy and associated factors among caregivers of children under 5 years old visiting Tibebe Ghion Specialized Hospital, Northwest Ethiopia, in 2023.

### 1.2 Specific Objectives

To determine the proportion of traditional uvulectomy among caregivers of children under 5 years old visiting Tibebe Ghion Specialized Hospital, Northwest Ethiopia, in 2023.

To identify factors associated with traditional uvulectomy among caregivers of children under 5 years old visiting Tibebe Ghion Specialized Hospital, Northwest Ethiopia, in 2023.

## 2 Methods

### 2.1 Study Design and Period

An institutional‐based cross‐sectional study was conducted among caregivers of children under 5 years old who visited Tibebe Ghion Specialized Hospital (TGSH) in Northwest Ethiopia. The study took place from October 1, 2023 to December 30, 2023.

### 2.2 Study Area

The study was conducted at TGSH, located in Bahir Dar, the capital of the Amhara region, Ethiopia, approximately 484 km northwest of Addis Ababa. The hospital established in 2010 E.C., TGSH is a specialized hospital and a teaching facility for undergraduate and postgraduate medical and health science students. It comprises nine departments: Pediatrics and Child Health, Internal Medicine, Surgery, Gynecology, Obstetrics, Dermatology, Orthopedics, Radiology, Ophthalmology, and ENT. Serving a catchment population of around 5 million, the hospital provides both outpatient and inpatient services and functions as a referral center for district primary hospitals and health centers.

### 2.3 Study Population

The source populations were all caregivers of children under 5 years old visiting TGSH, while the study populations were caregivers of children under 5 years old visiting the Pediatrics department during the study period.

#### 2.3.1 Inclusion Criteria

Caregivers with children under 5 years of age who were present and available to provide information at the time of data collection were included.

#### 2.3.2 Exclusion Criteria

Caregivers who were unaware of the child's uvulectomy status or who were not the child's parent (mother or father) were excluded from the study.

### 2.4 Variables of the Study

#### 2.4.1 Dependent Variable

The dependent variable for this study is Traditional Uvulectomy.

Traditional Uvulectomy (Yes = 1, No = 0).

#### 2.4.2 Independent Variables

The independent variables for this study include sociodemographic factors, maternal health service‐related factors, and awareness and belief‐related factors, as described below:

1. Sociodemographic factors: Place of residence, age of the child, sex of the child, parity of the mother, occupation of the parents, and educational status of the parents.

2. Maternal health service‐related factors: Place of delivery, antenatal care follow‐up, and postnatal counseling.

3. Awareness and belief‐related factors.

4. Awareness of the harmful effects of traditional uvulectomy: Whether the caregiver is aware of the health risks associated with traditional uvulectomy, including potential complications like infection and hemorrhage.

5. Perception of traditional uvulectomy: The caregiver's personal views or beliefs regarding the safety and effectiveness of traditional uvulectomy as a treatment for throat‐related issues.

6. Beliefs about the uvula causing illness: The caregiver's beliefs about the uvula's role in causing health problems, such as sore throat or respiratory issues, may influence their willingness to seek uvulectomy.

7. Beliefs about illnesses being curable by drug treatment: The caregiver's belief in the efficacy of pharmaceutical treatments for illnesses, may impact their choice of traditional versus modern medical interventions.

8. Observed positive outcomes following traditional uvulectomy: Whether the caregiver has witnessed favorable results, such as the resolution of symptoms like sore throat or coughing, after the traditional uvulectomy procedure.

### 2.5 Operational Definitions

1. Traditional uvulectomy: Surgical removal of the uvula by traditional practitioners. Measured based on caregiver history.

2. Harmful traditional practices: Includes practices such as female genital mutilation, traditional uvulectomy, milk teeth extraction, and traditional male circumcision.

3. Postnatal counseling: Counseling provided after childbirth.

4. Seeing previous good results after traditional uvulectomy: Witnessing resolution of complaints following uvulectomy.

5. Beliefs about the uvula causing illness: The caregiver's beliefs about the uvula's role in causing health problems, such as sore throat or respiratory issues, may influence their willingness to seek uvulectomy.

### 2.6 Sample Size Determination

The sample size for the study was calculated using a single proportion formula to estimate the proportion of caregivers practicing traditional uvulectomy. Using a proportion (*p*) of 52.5% based on prior studies [20], with a confidence level of 95% (*z* = 1.96) and a margin of error (*d*) of 5%, the sample size was initially calculated as 383. To account for potential nonresponse, we increased the sample size by 5%, resulting in a final sample size of 402.

For the second objective, sample sizes of 176 and 382 were estimated based on significantly associated variables from previous research. The larger sample size of 402 was chosen for the study to ensure sufficient power to detect meaningful associations while minimizing potential biases.

### 2.7 Sampling Technique

In this study, systematic sampling was chosen because mothers were selected during their visits to the hospital. More importantly, we were able to use it even though we had no sampling frame and assumed the population was homogeneous. Participants were selected with a sampling interval (*K*) of 2. Based on previous patient registration data, approximately 796 caregivers of children under five visited TGSH, yielding a *K* value of 2. This method ensured efficient participant selection, minimizing bias while maintaining a representative sample of caregivers visiting the hospital, allowing for reliable and comprehensive results.

### 2.8 Data Collection Tools and Procedure

Data were collected using an interviewer‐administered structured questionnaire which was divided into three sections: sociodemographic factors, maternal health service‐related factors, and awareness and belief‐related factors. The questionnaire was initially drafted in english, then translated into Amharic and back‐translated into English to ensure accuracy and maintain the integrity of the questions. The recruitment process involved selecting caregivers of children under 5 years old visiting the hospital for child care. Data collection was carried out by two trained nurses who were provided with standardized training to minimize interviewer bias. The nurses were under‐supervised by a senior nurse to ensure consistent data collection procedures. To further reduce bias, the interviewers followed a strict protocol and used neutral, non‐leading questions when administering the questionnaires. Each questionnaire was coded, and data were checked daily for completeness and consistency to ensure high‐quality data.

### 2.9 Data Quality Control

Data quality was ensured through pre‐data collection training for data collectors and supervisors, a pretest conducted on 5% of the sample size at Felege Hiwot Referral Hospital, Ethiopia, and subsequent adjustments were made. During data collection, supervisors closely monitored the process, and data were reviewed daily for accuracy and completeness.

### 2.10 Data Processing and Analysis

Data were cleaned, coded, and entered into Epi‐Data version 4.3, then exported to SPSS version 23 for analysis. Descriptive statistics (frequency, percentage, mean, SD) were used to describe participant characteristics. Results were presented in tables, graphs, and text. Binary logistic regression was used to assess associations between independent variables and the dependent variable. Variables with *p*‐values < 0.25 in the bivariable analysis were included in multivariable logistic regression analysis. Adjusted Odds Ratios (AOR) and 95% Confidence Intervals (CI) were calculated to determine the strength and precision of the associations. *p*‐values less than 0.05 and AOR with CI were used to determine statistical significance association. with a significance level set at *p* ≤ 0.05. Multicollinearity was assessed using tolerance and variance inflation factor (VIF). Model fitness was evaluated using the Hosmer and Lemeshow test, which resulted in a *p*‐value of 0.67, indicating that the model fits the data well.

### 2.11 Ethical Considerations

Ethical clearance was obtained from the Institutional Review Board of the College of Medicine and Health Sciences, Bahir Dar University, Ethiopia. Informed consent was taken from each participant following a detailed explanation of study objectives, Participants' rights, and confidentiality. Confidentiality was maintained throughout data collection, processing, and dissemination.

## 3 Results

### 3.1 Sociodemographic Characteristics

The study utilized a structured questionnaire consisting of questions that assessed socio‐demographic characteristics, practices, awareness, and beliefs toward traditional uvulectomy. The questionnaire covered a range of factors, including caregiver characteristics, the practice of traditional uvulectomy, caregivers' awareness, and their perceptions of its effects. this study adds a new dimension by analyzing factors such as maternal education, antenatal care (ANC) follow‐up, and caregivers' perceptions of the harmfulness of traditional uvulectomy. This is particularly important in understanding the underlying cultural and healthcare‐related factors influencing the practice.

The study included 402 caregivers of children under 5 years old. Of these, 203 (50.5%) were male and 199 (49.5%) were female. The age distribution of the children was as follows: 69 (17.2%) were under 6 months, 92 (22.9%) were between 6 and 12 months, 144 (35.8%) were between 12 and 24 months, and 97 (24.1%) were between 24 and 59 months. More than half of caregivers, 205 (51.0%), were rural residents, and 301 (74.9%) were multiparous. Regarding educational status, 206 (51.2%) of the mothers were illiterate. About, 204 (50.7%) of mothers and 248 (61.7%) of fathers were farmers. Most caregivers had a monthly income between 2000 and 5000 ETB (Table 1).

**Table 1:** Socio‐demographic characteristics of caregivers of children under five years at Tibebe Ghion Specialized Hospital, Bahir Dar, Ethiopia, 2023.

Variable	Category	Frequency (*n*)	Percent (%)
Child age (months)	< 6 months	69	17.2
	6–12 months	92	22.9
	12–24 months	144	35.8
	24–59 months	97	24.1
Sex of child	Male	203	50.5
	Female	199	49.5
Place of residence	Urban	197	49.0
	Rural	205	51.0
Mother**'**s age (years)	≤ 25	80	19.9
	25–29	134	33.3
	30–34	73	18.2
	≥ 35	115	28.6
Parity of mother	Primiparous	101	25.1
	Multiparous	301	74.9
Mother**'**s occupation	Housewife	63	15.7
	Civil servant	55	13.7
	Merchant	51	12.7
	Farmer	204	50.7
	Other	29	7.2
Father**'**s occupation	Daily Laborer	11	2.7
	Civil servant	75	18.7
	Merchant	55	13.7
	Farmer	248	61.7
	Other	13	3.2
Mother**'**s education	Cannot read/write	206	51.2
	Can read/write, no formal education	9	2.2
	Primary school	74	18.4
	High school and above	113	28.1
Monthly income (ETB)	< 2000	87	21.6
	2000–5000	190	47.3
	5000–8000	81	20.1
	> 8000	44	10.9

### 3.2 Practice of Traditional Uvulectomy

The proportion of traditional uvulectomy among caregivers of children under 5 years old was 46% (95% CI; 41–49). Of the children who underwent traditional uvulectomy, 150 (81.1%) were ill and 35 (18.9%) were healthy at the time of the procedure. Notably, 97.3% of the uvulectomies were performed on children under 6 months of age.

### 3.3 Caregivers' Awareness and Belief Toward Traditional Uvulectomy

Among all caregivers, 254 (63.2%) believed that the uvula causes one or more illnesses in children. The most commonly perceived illnesses were sore throat, fever, swallowing difficulty, behavioral change, and vomiting. More than half of the respondents, 224 (88.2%), were aware that these illnesses could be treated with modern medicine. However, 275 (68.4%) of the respondents had not seen anyone with good health after a traditional uvulectomy. Of the caregivers who practiced traditional uvulectomy, only 53 (28.6%) had information about its harmfulness, and 143 (77.3%) believed that the uvula could cause one or more illnesses. The most frequently reported illnesses caused by the uvula were sore throat (120, 64.8%), swallowing difficulty (117, 63.2%), and fever (90, 48.6%) (Table 2).

**Table 2:** Awareness, perception, and maternal‐related factors of caregivers of children under five years at Tibebe Ghion Specialized Hospital, Bahir Dar, Ethiopia, 2023.

Variable	Category	Traditional uvulectomy (Yes)	Frequency	Percent (%)
A belief that Uvula causes illness	Yes	254	254	63.2
	No	148	148	36.8
If yes, what disease it causes	Sore throat	233	233	57.9
	Fever	177	177	44.0
	Swallowing difficulty	219	219	54.4
	Behavioral change	66	66	16.4
	Vomiting	67	67	16.6
	Other	17	17	4.2
A perception that this illness can be cured with modern treatment	Yes	224	224	88.2
	No	30	30	11.8
Information about the harmfulness of uvulectomy	Yes	173	173	43.0
	No	229	229	57.0
Have you seen anyone in good health after a uvulectomy?	Yes	127	127	31.6
	No	275	275	68.4
Place of delivery	Home	37	37	75.5
	Institution	148	148	41.9
Antenatal care follow‐up	Yes	106	106	36.2
	No	79	79	72.4
Counseling about traditional uvulectomy	Yes	19	19	16.8
	No	166	166	57.4

### 3.4 Maternity Related Factors

Around 353 (87.8%) of the mothers gave birth at a health facility, and only 293 (72.9%) of them had ANC follow‐up during their pregnancy. Of all the respondents who practiced traditional uvulectomy, 148 (80.0%) of them delivered at a health facility, and 106 (57.3%) had ANC follow‐up. However, only 19 (10.3%) of them received counseling about traditional uvulectomy practice during the postnatal period (Table 2).

### 3.5 Factors Associated With Traditional Uvulectomy

We employed both bivariable and multivariable binary logistic regression analyses to identify factors significantly associated with the practice of traditional uvulectomy. Bivariable logistic regression was initially used to select candidate variables, while multivariable logistic regression was then applied to adjust for potential confounding factors. The model's goodness of fit was assessed using the Hosmer and Lemeshow test, which resulted in a *p*‐value of 0.67, indicating that the model fits the data well.

In the bivariable analysis, several factors were associated with traditional uvulectomy at a significance level of *p* < 0.25. These factors include the age of the child, sex of the child, residence, maternal educational status, paternal educational status, place of delivery, antenatal care (ANC) follow‐up, the belief that the uvula causes illness, awareness of traditional uvulectomy and having observed a positive outcome after traditional uvulectomy.

In the multivariable logistic regression analysis, five variables remained significantly associated with the practice of traditional uvulectomy at *p* < 0.05: maternal educational status, place of residence, place of delivery, ANC follow‐up, and the belief that the uvula causes illness.

Caregivers who are unable to read and write were 1.85 times (AOR: 1.85: 1.39–6.27) more likely to have traditional uvulectomy as compared to caregivers who are able to read and write. Caregivers from rural areas were 2.81 times (AOR: 2.81: 1.63–5.68) more likely to practice traditional uvulectomy than caregivers from urban areas. caregivers who had no ANC follow‐up were 5.20 times (AOR: 5.20: 2.06–8.63) more likely to engage in traditional uvulectomy than caregivers who had ANC follow‐up. Additionally, caregivers who had no awareness about traditional uvulectomy were 2.43 times (AOR: 2.43: 1.24–4.55) more likely to practice it compared to those with prior awareness. finally, caregivers who had witnessed positive outcomes from traditional uvulectomy were 6.05 times more likely to practice it than those who had not observed such results (AOR: 6.05: 3.64–12.11) (Table 3).

**Table 3:** Factors associated with traditional uvulectomy practice of caregivers of children under five years at Tibebe Ghion Specialized Hospital, Bahir Dar, Ethiopia, 2023.

Variable	Category	Yes (*n*)	No (*n*)	COR (95% CI)	AOR (95% CI)	*p*‐value
Age of child (months)	< 6 months	28	41	1.06 (0.56, 1.99)	1.01 (0.43, 1.67)	0.472
	6–12 months	48	44	1.69 (0.95, 3.01)	1.25 (0.62, 2.94)	0.337
	12–24 months	71	73	1.51 (0.89, 2.54)	1.17 (0.41, 2.08)	0.144
	> 24–59 months	38	59	1	1	
Sex of child	Male	100	103	1.30 (0.87, 1.92)	1.68 (0.94, 3.26)	0.521
	Female	85	114	1	1	
Maternal education	Can't read/write	107	99	1.63 (1.10, 2.42)	1.85 (1.39, 6.27)	0.023
	Can read/write	78	118	1	1	
Paternal education	Can't read/write	81	76	1.44 (0.96, 2.16)	1.29 (0.47, 4.76)	0.506
	Can read/write	104	141	1	1	
Place of residence	Rural	104	101	1.47 (0.99, 2.18)	2.81 (1.63, 5.68)	0.001
	Urban	81	116	1	1	
Delivery place	Home	37	12	4.27 (2.15, 8.46)	2.02 (0.92, 3.57)	0.149
	Institution	148	205	1	1	
ANC follow‐up	No	79	30	4.64 (2.86, 7.53)	5.20 (2.06, 8.63)	< 0.001
	Yes	106	187	1	1	
Belief that uvula causes illness	Yes	143	111	3.25 (2.10, 5.02)	1.54 (0.70, 3.43)	0.432
	No	42	106	1	1	
Information about the harm caused by traditional uvulectomy	No	132	97	3.08 (2.03, 4.67)	2.43 (1.24, 4.55)	0.013
	Yes	53	120	1	1	
Saw good results after traditional uvulectomy	Yes	102	25	9.43 (5.68, 15.67)	6.05 (3.64, 12.11)	< 0.001
	No	83	192	1	1	

## 4 Discussion

In our study, the proportion of traditional uvulectomy was 46%. This result was comparable to a study conducted in the South Gondar Zone, Ethiopia (52.5%) [20]. The finding was lower than that of studies conducted in Axum town, Ethiopia (86.9%), and Nigeria (68.8%) [21]. The difference might be due to the study setting, where the study in Axum was community‐based, and cultural disparities between the studies in Nigeria, where uvulectomy is considered as religious dictate. However, the finding was higher than studies conducted in Debre Tabor, Ethiopia (15.88%), Democratic Republic of the Congo (18.7%) and Niger (19.6%) [8, 10, 18]. This might be due to differences in the study period, socioeconomic factors, healthcare infrastructure, and educational campaigns in shaping cultural practices related to healthcare.

Caregivers from rural residents were 2.81 times more likely to practice traditional uvulectomy (AOR: 2.81: 1.63–5.68) than urban residents. Similarly, in a study done in Southern Ethiopia, participants who were rural residents were nearly five times more likely to perform harmful cultural practices [22]. This could be because rural areas often lack access to healthcare services, leading to higher reliance on traditional practices. Also, cultural beliefs and practices tend to be stronger in rural communities, which could contribute to the persistence of traditional uvulectomy.

Caregivers who could not read and write were 1.85 times (AOR: 1.85: 1.39–6.27) more likely to practice traditional uvulectomy. This is in line with a study conducted in Nigeria where women with no formal education practice traditional uvulectomy than those with formal education [23]. This could be due to limited access to health information and lower awareness of the negative consequences of traditional uvulectomy.

In addition, caregivers who had no ANC follow‐up were 5.20 times (AOR: 5.20: 2.06–8.63) more likely to practice traditional uvulectomy. A similar study in Awi zone, Ethiopia, found that women who had lower levels of education and those living in rural areas were less likely to have received ANC and were more likely to practice harmful traditional practices [24]. This could be because women who had no ANC follow‐up will miss counseling on the drawbacks of traditional uvulectomy and the benefits of health care seeking during a child's illness.

Moreover, caregivers who had no information about the harmfulness of the practice (AOR: 2.43: 1.24–4.55) and saw good results after the procedure (AOR: 6.05: 3.64–12.11) were more likely to practice traditional uvulectomy, which is consistent with earlier studies conducted in Northwest, Ethiopia [24]. This could be due to the information gap and acceptance of the practice as an effective and alternative option.

### 4.1 Limitations

Since the study was facility‐based it may not represent the prevalence of traditional uvulectomy in the general population.

## 5 Conclusions

The proportion of traditional uvulectomy remains high in the study area, and it is associated with factors such as illiteracy, lack of ANC follow‐up, rural residency, lack of awareness about the harmful effects of uvulectomy, and witnessing positive outcomes. Immediate actions should include increasing awareness through ANC counseling and educating caregivers on the risk of uvulectomy. Long‐term solutions could involve policy changes and community‐based interventions aimed at reducing the practice and its associated risks.

## 6 Recommendations

Based on the findings of this study, the following recommendations are proposed:

Health care facility: should strengthen awareness campaigns within the health facilities regarding the effects of traditional uvulectomy. Provide comprehensive counseling during antenatal and postnatal visits focusing on the dangers of this practice and the importance of modern healthcare alternatives for child health.

Community health workers: should identify traditional practitioners who perform traditional uvulectomy within the community and provide them with education on the risks associated with this practice. Additionally, they should work to inform caregivers about safer alternatives and promote awareness of the harmful consequences of traditional vulvectomy.

Community leaders: should play a key role in addressing traditional uvulectomy by engaging with local populations to expose and discourage harmful practices. They should collaborate with health workers to educate caregivers about the risks of traditional uvulectomy and advocate for healthier practices.

Future researchers: Future studies should include large‐scale community‐based and qualitative research to explore the cultural, socioeconomic, and healthcare factors. This will help to develop more effective interventions that target both the practice and its underlying causes.

## Author Contributions

**Gebiyaw Wudie Tsegaye:** conceptualization, methodology, software, writing – original draft, formal analysis, and supervision. **Martha Tuji:** conceptualization, visualization, funding acquisition, investigation, validation, and data curation. **Shitahun Fentie:** supervision, project administration, visualization, investigation, methodology, and software.

## Ethics Statement

Ethical clearance was obtained from the Institutional Review Board of the College of Medicine and Health Sciences, Bahir Dar University. Informed consent was secured from each participant after explaining the study objectives. Confidentiality was maintained throughout data collection, processing, and dissemination.

## Conflicts of Interest

The authors declare no conflicts of interest.

## Transparency Statement

The lead author Gebiyaw Wudie Tsegaye affirms that this manuscript is an honest, accurate, and transparent account of the study being reported; that no important aspects of the study have been omitted; and that any discrepancies from the study as planned (and, if relevant, registered) have been explained.

## Data Availability

The data were collected from an individual caregiver who visits TGSH. The collected information was used only for research purposes and was kept confidential. The corresponding Author will provide the data set used and or analyzed during the study upon responsible request.
